# The role of improved housing and living environments in malaria control and elimination

**DOI:** 10.1186/s12936-020-03450-y

**Published:** 2020-10-31

**Authors:** Richard Carter, Nadira D. Karunaweera

**Affiliations:** 1 School of Biological Sciences, University of Edinburgh, Edinburgh, UK; 2 Department of Parasitology, Faculty of Medicine, University of Colombo, Colombo, Sri Lanka

**Keywords:** Malaria transmission, Malaria control, Malaria elimination, Housing, Ross/Macdonald equations, Reproduction number, Sri Lanka, Living environment, Socio-economic development

## Abstract

Malaria risk and endemicity is often associated with the nature of human habitation and living environment. The disappearance of malaria from regions where it had been endemic for centuries, such as coastal areas of southern England, has been attributed, at least in part, to improvement in the quality of housing. Moreover, indigenous malaria transmission ceased throughout England without the necessity to eliminate the vector mosquitoes. The principles of malaria transmission, as formulated following the thinking of the pioneers of malaria epidemiology, Ronald Ross and George Macdonald, show how this may happen. Malaria ceases to be sustainable where its reproduction number, R0, the number of new cases generated on average for each existing case of malaria, falls below 1. In the terms of a Ross/Macdonald analysis the reduced contact between humans and blood-feeding mosquitoes that is achieved through housing that is secure against mosquito entry can have a powerful effect in reducing malaria R0. The island of Sri Lanka, where malaria had been endemic probably for centuries previously, has reported no indigenous cases of malaria since 2012. The disappearance of malaria from Sri Lanka followed an effective attack upon malaria transmission by the Sri Lanka Anti Malaria Campaign. The targeted and enhanced efforts of this campaign launched in 1999, drove the malaria R0 below 1 for most of the period up to 2012, leading to a nearly continuous decline in malaria cases until their extinction. The decades leading up to the launch of these efforts were ones of general improvement of living environment and notably in the quality of housing stock. Studies in the late 1980s had shown that quality of housing in a highly malarious district of Sri Lanka was a strong determinant of malaria risk. Through its effects on malaria R0, improved housing is likely to have facilitated the malaria control and cessation of indigenous malaria transmission in Sri Lanka and that it will help reduce the risk of the re-introduction of malaria to the island.

## Background

For the period of written history, and probably long before it, the nature of human habitation and the man-made environment has influenced the presence or absence of malaria transmission [1, 2]. In recent decades there has been renewed interest in this association [3–6] driven by awareness that better general standards of living and of housing tend to mitigate against malaria transmission. Here, the relationship between housing and living environment and the disappearance of malaria in a historical example in England and a recent example in Sri Lanka is discussed.

## The disappearance of malaria from England

English wetlands, much of them southern coastal salt marsh, which had been highly malarious since at least late medieval times [7], became effectively malaria-free in the first decades of the 20th Century [8]. Between 1917 and 1926, as malaria-infected soldiers returned from the First World War, malarial infections re-appeared among local inhabitants across these same areas of England [8]. However, no further transmission took place. Even though the malaria vector mosquitoes (e.g.,*. Anopheles atroparvus* and *Anopheles plumbeus* [9]) were clearly still present and competent to generate new cases from introduced ones, the previously malarious regions of England had apparently become incapable of sustained malaria transmission.

How could this be? The answer, James [8] argued, lay to a large extent in two transformations. One was that by the early 20th Century the anti-malarial drug, quinine, had become widely affordable and available in England. The other lay in the quality of the human living environment, and above all of human dwellings. James describes it thus. In contrast to dwellings of *“straw or stones or mud bricks, without windows or means of introducing light and ventilation….invariably infested with anopheles mosquitoes…In England… “civilising” social influences…particularly during the last seventy years (i.e. since about 1860) … (have resulted in) houses (that) are better lighted and ventilated; they have windows and are less damp; they have floors and are provided with ceilings shutting off the bedrooms from the rafters of the roof, they are more open and less crowded and are more frequently painted and whitewashed on the inside than they used to be. These changes, as well as more cleanly conditions in the home generally, have made the houses much less liable to harbour anopheles mosquitoes and have broken, to a considerable extent, the close association between those mosquitoes and man which existed when living conditions were primitive. Undoubtedly this disassociation has contributed materially towards the reduction of malaria.”* The idea that James espoused as a major component to the disappearance of malaria from England was not the total elimination of the vector mosquitoes (important as their numerical reduction would have been through drainage of wetland [10]) but the sufficient reduction in contact between these mosquitoes and their human hosts through decent housing. Reduction in human and *Anopheles* contact had also occurred through increase in the cattle population as diversionary hosts to the mosquitoes [10].

## Principles of malaria transmission and the human living environment

James’ ideas are well supported by the theoretical principles of malaria transmission. Pioneered by Ronald Ross [11] they were formulated by George Macdonald [12–14] in terms that, although subject to ongoing analysis and modification, are still broadly accepted. A central concept presented by Macdonald is that of the “*basic reproduction number for malaria*”—the number of new cases resulting from each existing case of malaria—now designated R0, is given in what is widely known as a Ross/Macdonald equation [14]. In such an equation (e.g., Box 1) R0 is, among other factors, a function of ‘M’, the number of adult female malaria vector mosquitoes in a defined locality, and of ‘a’, their daily biting rate upon humans. Reducing either or both M and a reduces R0. Because R0 is proportional to a^2^ (Box 1), anything that reduces a, the daily rate at which vector mosquitoes take a human blood meal, is particularly powerful in reducing the value of R0. Improved house-type construction that is secure against mosquito entry reduces a. It is likely that there are other malaria transmission-reducing effects that result from those types of housing that resist entry by mosquitoes. These include their impact upon mosquito egg-laying rates due to the lower frequency of blood meals. Recent analysis indicates that such effects on M (Box 1) could also significantly reduce R0 [15]. Improvements in housing are, therefore, as James proposed, likely to have contributed greatly to the reduction leading to disappearance of indigenous malaria transmission in England.

Box 1 A Ross/Macdonald equation The following formulation is given of a Ross/Macdonald equation:

R0, as defined by Macdonald (see below) is “*The number of infections distributed in a community as a direct result of a single primary non*-*immune case*”. In the case of malaria he proposed the following relationship between R0 and its determining factors:

R0 = (M/H)a^2^.c.b.p^v^/(−lnp.r)

M is the density (e.g., numbers per sq km) of adult female vector *Anopheles* mosquitoes in a (defined) locality;

H is the density of humans (i.e., numbers per sq km) in this locality;

a is the proportion of female mosquitoes that feed on humans each day;

c is the proportion of blood meals on an infected human that will yield an infection in the mosquito;

b is the proportion of bites by an infectious mosquito that infect a human;

p is the daily probability of survival of a mosquito;

v is the time in days from a human blood meal until a mosquito becomes potentially infectious to a human;

r is the daily rate at which each infected human completely clears infection.

As pointed out in the text, the probability that an adult female mosquito takes a human blood meal, ‘a’, has a powerful effect upon R0 because it is squared in the equation for R0. For example reducing a by a half reduces R0 by a quarter; reducing a by 90% reduces R0 to 1% and so on. Compared to many traditional forms of rural housing in Sri Lanka, modern housing with sealed doors, windows, eaves, etc. and interiors with fewer resting places for mosquitoes, reduces the human biting rate by mosquitoes, a, many fold. In addition, reducing the frequency of mosquito blood meals proportionately reduces their egg-laying rate and hence the numbers of adult female mosquitoes ‘M’.

The Ross/Macdonald equation, presented here, derives from that given by George Macdonald in 1952 [12] when he put forward the concept of a reproduction number for malaria. Indeed, this appears to have been the first statement of the idea of a reproduction number, R0 (which he called Z0 at the time), for any infectious disease. As he would point out [13] “*Should this (number, i.e. R0) fall below one, successive generations of cases would be smaller than their predecessors and the disease would disappear; should it be greater than one, successive generations would increase and the disease would mount in the population*.”

## The termination of autochthonous malaria transmission in Sri Lanka

**Fig. 1:**
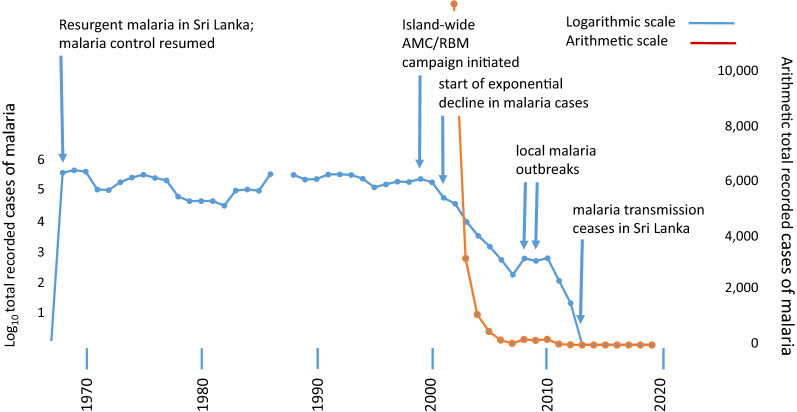
Recorded cases of indigenously transmitted malaria in Sri Lanka, 1967 to 2020 (Source: Anti Malaria Campaign, Ministry of Health, Sri Lanka) (AMC: Anti-malaria Campaign; RBM: Roll Back Malaria). logarithmic scale blue lines; arithmetic scale red lines

**Fig. 2:**
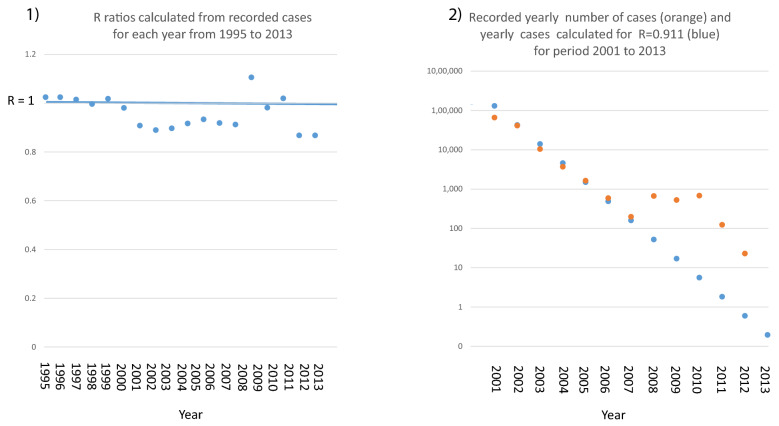
Panel 1 R ratios calculated from recorded cases for each year from 1995 to 2013. R ratio is below 1 for the years 2001 to 2007 and again for the years 2011 and 2012. In the years 2008 and 2009 there were two local outbreaks of malaria leading to the island wide R rising to 1 or above for the years 2008 to 2010. Panel 2 Recorded yearly number of cases (orange) and yearly cases calculated for R = 0.911 (blue) for period 2001 to 2013

When the malaria R0 is reduced to a stable value below 1, irrespective of the cause, then malaria incidence can be expected to decline continuously and exponentially. These expectations are well met by the recorded cases of malaria in Sri Lanka for most of the period from 2001 to 2012 (Fig. 1) [16, 17] (Fig. 2). In 2013 no indigenously acquired case of malaria was recorded in Sri Lanka. There have been none since [17–19] except for a recent case acquired by infection from a foreign migrant [20].

Fig. 2 R and the decline of malaria transmission in Sri Lanka Let ‘R’ = (number of new cases after one round of malaria transmission)/(number of cases at the start of this round of transmission).

Let ‘n’ be the number of cases at the start of this round of transmission.

After one round of transmission, the number of new cases = n.R, after two rounds of transmission = n.R.R = n.R^2^ after three = n.R^3^ and so on. Where R is fewer than 1, this progression is a simple exponential decline.

The rate of decline of malaria cases in Sri Lanka from 2001 through 2007 was about two-thirds per year. Based on the above progression and assuming there are 12 rounds of malaria transmission per year, then from 2001 to 2007 this corresponds to an almost stable R = 0.91 per round of transmission (panel 1) leading to an exponential decline that matches very closely with the recorded decline in malaria cases during this period (panel 2). With the exception of local malaria outbreaks in 2008 and 2009 and their subsequent suppression, it continued to the termination of indigenous malaria transmission on the island of Sri Lanka in 2013 (Fig. 2).

It is pointed out that the evidence quoted here on house type and malaria [22, 23] concerns malaria risk. This is not a measure of malaria transmission rate. However, the factors that influence malaria risk, up or down, closely overlap those (e.g., human biting rate) that influence the case reproduction number, R.

Thus, an island-wide tipping point for malaria transmission (R0 < 1) appears to have arisen at around the year 2000 sending malaria case incidence into its exponential decline (Fig. 1, Box 2). It coincides with the enhanced malaria control in endemic areas begun in 1999 by the Sri Lanka Anti Malaria Campaign in line with the Roll Back Malaria initiative of the World Health Organization [19]. The effectiveness of this campaign, which involved targeted and coordinated interventions against multiple components of malaria transmission in the human host and in the mosquito vectors, can be understood, in part, in terms of the Ross/Macdonald equation (Box 1).

**Fig. 3:**
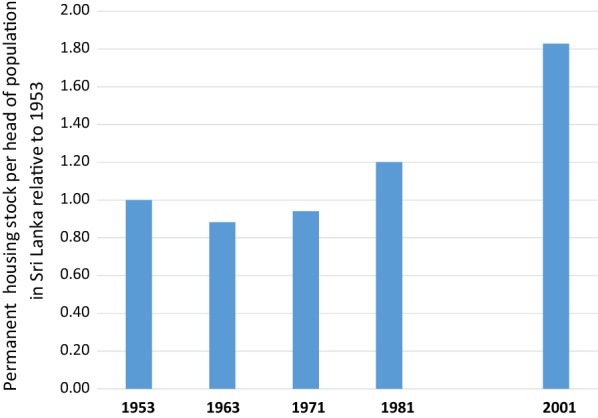
Permanent housing stock per head of population in Sri Lanka relative to values in 1953. Calculated from data in Table 3.5 in Housing and Sustainable Urban Development in Sri Lanka. National Report for the Third United Nations Conference on Human Settlements Habitat III. 2015. http://habitat3.org/wp-content/uploads/Sri-Lanka-%EF%BC%88Final-in-English%EF%BC%89.pdf. No census data available for 1991 due to the armed conflict that prevailed during that period

**Fig. 4:**
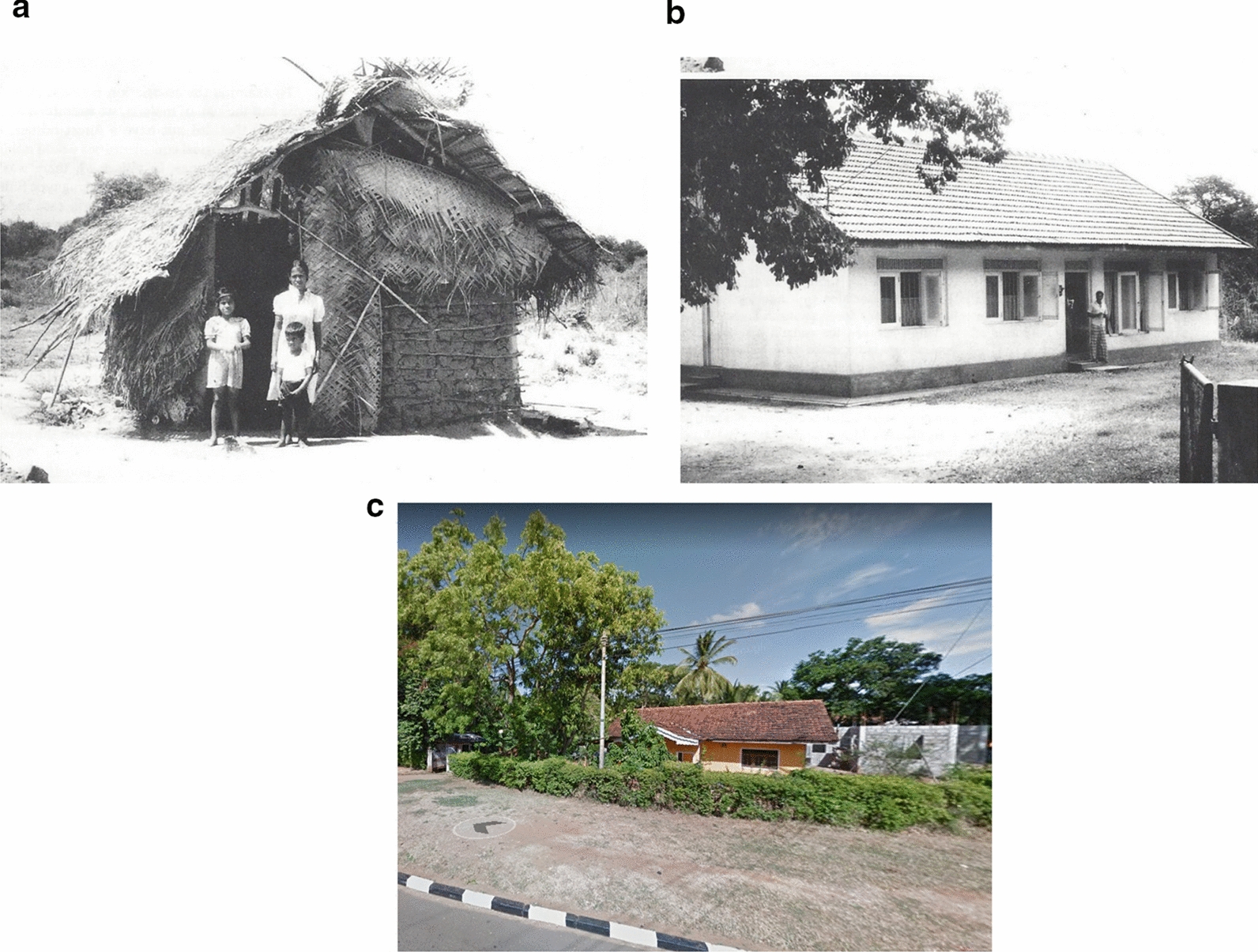
Housing in Kataragama, Moneragala District, Sri Lanka, from around 1990 and in 2018. **a**, **b** Kataragama, Moneragala District (circa 1990). **c** Kataragama, Moneragala District (2018)

Its success would also have been facilitated by the steadily improving human–environment, and specifically better housing conditions across the island in the preceding decades [21] (Figs. 3 and 4). A strong association between better type of house construction and reduced malaria risk had indeed, been demonstrated in studies conducted in the late 1980s and early 1990s in Moneragala [22, 23], a district of Sri Lanka that was at the time highly malarious. The results of these studies are a direct demonstration of the relationship between good housing and reduced malaria as proposed by James [8]. The trend towards better housing in Sri Lanka since the 1980s would undoubtedly have created an ever-more malaria transmission-resistant environment within which to conduct active malaria control.

While caution and vigilance should be maintained against the possible return to endemic malaria in Sri Lanka [24], continuing socio-economic improvements in malaria-prone areas, including in housing and life style, will help mitigate against its resurgence. Such improvements represent national economic and social development as significant forces in assisting malaria control and prevention.

## Data Availability

Not applicable.

## Abbreviations

R0: Reproduction number
M: Number of adult female malaria vector mosquitoes in a defined locality
a: Daily mosquito biting rate upon humans
